# Factors Influencing Formal Mental Treatment - Seeking Behaviour among Caretakers of Mentally Ill Patients in Zanzibar

**DOI:** 10.24248/eahrj.v6i2.694

**Published:** 2022-11-30

**Authors:** Said S. Bakar, Fabiola M. Moshi

**Affiliations:** a Department of Clinical Nursing, School of Nursing and Public Health, the University Dodoma; b Department of Nursing Management and Education, School of Nursing and Public Health, the University Dodoma

## Abstract

**Background:**

Mental illnesses are health conditions which are associated with changes in emotion, thinking, or behaviour (or a combination of these). Healthcare-seeking behaviour for formal mental health treatment is lacking all over the world, particularly in low and middle-income countries. Inappropriate health-seeking behaviours are reported to result in delays in seeking appropriate care and thus increase the risk of complications in mentally ill patients. The study aimed to assess factors influencing formal mental treatment-seeking behaviour among caretakers of mentally ill patients in Zanzibar

**Methods:**

A community-based cross-sectional study design was conducted from January to June, 2021. A total of 246 caretakers of mentally ill patients were recruited for the study using multi-stage sampling technique. An interviewer-administered semi-structured questionnaire was used to collect information from caretakers. Bivariate and multivariable logistic regression models were applied to determine the factors influencing formal mental treatment-seeking behaviour.

**Results:**

Majority of caretakers 187(76%) were aware of formal mental treatment. Also, majority of the participants 145(58.9%) had appropriate healthcare-seeking behaviour toward formal mental treatment. Factors influencing formal mental treatment-seeking behaviour were; perceived severity (AOR 4.651 at 95% CI 2,397-9.021 *p<.001*) and being aware (AOR 2.907at 95% CI 2.349-2.326 *p=.004*).

**Conclusion:**

Majority of caretakers were aware of formal mental illness treatment. Also, more than half of the caretakers had appropriate healthcare-seeking behaviour. Factors associated with formal mental treatment-seeking behaviour were awareness of formal mental treatment and perceived severity of mental illness. The study recommends a community sensitisation campaign to raise community awareness and perception towards formal mental treatment. Community sensitisation is crucial for improving formal mental treatment-seeking behaviour.

## BACKGROUND

Mental illnesses remains among the top 10 leading cause of burden worldwide with no sign of reduction since 1990.^1^ The statistics have estimated that there were 970^1^ million people around the globe who were diagnosed with mental illnesses in 2019.^2^ The number drastically increased in 2020 due to the coronavirus disease of 2019 (COVID-19) pandemic.^1^ Sub-Saharan Africa is reported to have at least 10% of the global mentally ill patients.^2^ East African countries form part of the sub-Saharan African countries with high proportion of people living with mental illness. In Kenya, 1 in 10 people is suffering from a common mental illness and the country ranks 5^th^ among African countries with the highest number of depression cases.^2^ Depression and anxiety are the main mental health illness in Uganda affecting 1 in 4 people.^3^ In Tanzania, mental illness contributed to disability-adjusted life years at 2,727.86 per 100,000 population in 2017.^2^ The prevalence of participants who had previously been diagnosed with mental illness in Zanzibar was found to be at 1.6% (CI 0.9-2.9) with more than half (25 out of 39 diagnosed) of them being diagnosed with anxiety.^4^ This big number of affected people and the associated impacts of mental illness motivate intervention. Empirical evidence shows that mental illness results in tremendous mortality, morbidity, and impairment.^2^ The World Health Organization (WHO) has estimated that 25% of the global population will suffer from atleast one type of mental illness in their lifetime.^1^

It is recognised that effective prevention and treatment of mental illness options do exist.^5^ In recognising this, the Government of Tanzania intentionally provides mental illness treatment services free of charge. Contrarily, there are many people with serious mental illnesses in the country but they do not attend treatment. It is argued that the associated stigma and irrelevant controls are contributing factors to the withdrawal of seeking mental illness treatments.^6^

Mental health policy is an important tool to improving the country's mental healthcare services. Mental health policy is a written policy document by the government's Ministry of Health that stipulates the goals for improving mental health in the country, priorities, and the directions to attain them.^7^ The policy may also include; advocacy for mental health goals, promotion of mental well-being, prevention of mental disorders, treatment of mental disorders, and rehabilitation mechanisms to help mentally ill clients to achieve optimum social and psychological functioning.^8^ Zanzibar introduced its first mental health policy in 1999. The implementation of the policy has improved mental health services for over 10 years.^9^

Most studies conducted within African communities revealed that mental illness is associated with supernatural causes, such as; curses, witchcraft, demons and God's will, and that the first point for seeking help is through traditional and religious healers.^10–12^ Formal treatment is usually considered the last option.^10^ The reported factors which influence formal mental health-seeking behaviour include; knowledge regarding mental illness; traditional beliefs; stigma and discrimination, knowledge about how to access treatment, and the perceived side effects of antipsychotic medication.^10,13^ People of Zanzibar, like other African communities seek alternative treatment for mental illness (traditional and spiritual healers) first and turns to formal treatment when the health status of their patients worsen.^14^ Delayed seeking of formal treatment has negative consequences for the individual, their family, the community, and the health system.

Mental illness affects the individual's ability to make an informed choice regarding his/her health. Place for treatment is mostly decided by a close relative. In this study, these close relatives are termed as informal caretakers. An informal caretaker is a person who provides healthcare or assistance to a friend or family member with a health problem or disability without payment and has no specialised training. Family support is a key predictor for formal mental health treatment among mentally ill patients.^15^

Formal treatments are treatments offered by trained professionals who utilise available resources to provide evidence-based treatments to people with mental illness.^5^ Being unaware of mental illness is the leading reason for disconnection between actual treatment and care-seeking tendency which, consequently makes people withdraw from seeking mental health services ordrop out the mental health service.^3^

The perception that mental illness is caused by supernatural powers is not the only barrier to formal mental health utilisation in Zanzibar, accessibility of mental health services in the region is also a contributing factor.^14^Studies indicate that negative perception of mental illness, such as; fear of stigma influence mentally ill patients and their caretakers' decision to withdraw from seeking health services.^6^

Health service physiognomies are illustrative factors related to the performance of proper and effective healthcare program. Among others, these are reliability, availability, and accessibility of drugs, quality medical care, and the attitude towards people with mental illness. Educational qualifications are categorised by the person's level of education accomplishment.^4^ The healthcare-seeking tendency is any action or inaction done by people who consider themselves to be having a health problem.^4^

The most barriers to care-seeking and service participation in mental illness are behaviours and perceptions that hinder health decisions. They involve stigma which contributes to avoidance of treatment or dropping out prematurely, poor understanding of mental illness issues, misinformation about the effectiveness of treatment, and lack of support networks that promote care-seeking and perceived cultural irrelevance of many treatments.^3^

Mental health services in Zanzibar are provided at Primary Health Care level (PHC) units in areas such as; Nungwi, Jambiani, Matemwe, Pwani Mchangani, Kitogani, Donge, Kiwengwa, Chaani, and Kikobweni. Secondary-level mental health services are provided in the district hospitals, these have a psychiatric clinic. There is one tertiary hospital, Kidongochekundu Mental Hospital, this provides outpatients and admission psychiatric services. The ultimate goal for this study was to determine factors associated with formal mental health-seeking behaviour among caretakers of mentally ill patients.

## METHODS

### Study Design and Setting

The study was a community-based analytical cross-section study conducted in Zanzibar. Zanzibar lies 25 miles off the East African coast. It is part of the United Republic of Tanzania and is comprised of 2 main islands, Unguja (1,464km^2^) and Pemba (868km^2^). The 2 islands are surrounded by numerous other islands along the East African coast. Zanzibar has 5 regions; Urban West, North and South Unguja, and North and South in Pemba. Zanzibar has one Mental hospital known as Kidongochekundu hospital located in Urban West region in Unguja island, and other psychiatric clinics in Unguja are Kivunge and Makunduchi District Hospitals, Komben, Kitogani, Upenja, and Donge PHC units.

Pemba Island has three District hospitals; Abdallah Mzee Hospital located in Mkoani District, Chake Chake hospital located in ChakeChake District (both in the South Region) and Wete hospital located in Wete District, North Region. The Island also has 2 Primary Health Care Centres (PHCC), located in Vitongoji-South Region and in Micheweni-North Region. The hospitals and health centres have more number of admitted psychiatric cases (outpatient departments in Kidongo Chekundu and the Psychiatric Units).

### Study Population

The study included caretakers of mentally ill patients in Zanzibar. Inclusion criteria was all caretakers above 18 years old who are caring for registered mentally ill patients in the Zanzibar community who were willing to participate, with no history of mental illness and having provided care to a mental illness patient for at least 6 months. Caretakers with history of progressive chronic physical illness and those who refused to participate in the study were excluded.

### Sample Size Estimation

Sample size of 246 respondents was obtained from a total study population of 5,298 using the formula:

n = Z^2^ p (1-p)/E^2^

Whereby:

n = sample size required

p = proportion 20% of people who use appropriate health-seeking behavior (17).

Z = Z-Score (1.96)

E = Marginal Error (5% = 0.05)

n = 1.96 × 1.96 × (1-0.2)/0.05 × 0.05 = 246

Therefore, a total of 246 respondents were recruited for the study.

### Sample Technique

Multistage random sampling procedure was used to select areas for data collection and study participants. Determining sample size was obtained by using multistage sampling involving several stages as shown in the figure (Adam Hayes, 2020).

The first stage was used to select a region; whereby 3 regions (1 in Pemba and 2 in Unguja) were involved in the study. Purposive sampling technique was utilised for selection of the regions. The second and third stages utilised Simple Random Sampling lottery technique to select Districts and Shehias within the 3 regions that were nominated for the study's data collection process. Caretakers were drawn from the register of mental illness patients at the respective unit that offers mental health services in Zanzibar. Patients' files were traced at the mental health unit to get the location of their homes and the contact of the responsible caretaker.

**FIGURE 1:**
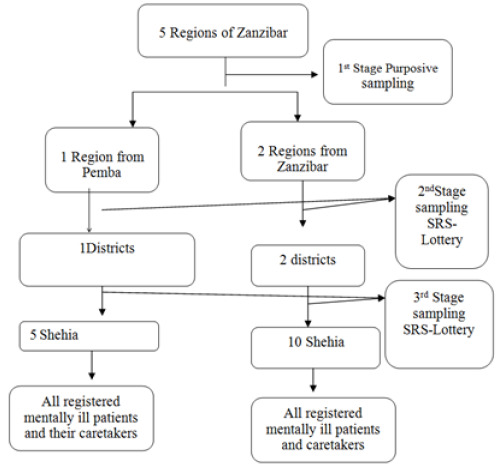
Represent summary of Sampling procedure and Technique

Systematic random sampling procedure was used to select Shehias in 2 regions of Unguja. The name of each Shehia was listed on a piece of paper and categorised into 5 groups of 24 names in each group. This gives chances for each district and Shehias to be picked randomly after getting a random number through the lottery method. The Shehias names used for data collection in Unguja were; Mlandege, Amani, Kwabinthamrani, Mtopepo and Nyamaz. For Northern region of Unguja and Southern region of Pemba, each has two districts. The lottery procedure was done separately for each region. This enabled mechanism for establishing a random number that allowed the selection of 5 Shehias per region. A systematic sampling procedure was used as mentioned above. Data was collected from the two regions in Shehias; Ole, Mkoroshoni, Shungi, Mfikiwa, Kengeja, and Uweleni, of the south region of Pemba and Kidoti, Nungwi, Mkokotoni, Kivunge, and Donge of the north region in Unguja as shown in Figure 1.

### Data Collection Methods, Tools and Procedures

Data was collected using semi-structured interview technique. The principal researcher and research assistants interviewed respondents and filled in the questionnaire appropriately as per response received. Collected data covered to answer all specific objectives, requirements and social demographic characteristics of caretakers. Research assistants were trained about the research aim, confidentiality, the data collection process, and on how to fill out the questionnaire. Questionnaires were standardised with a structure of closed-ended questions. Questions were translated into Kiswahili language for easy understanding by those who wished to read the questionnaire by themselves.

Documentary review was used to assess the outpatient mental ill registration register. Relevant information was collected. This included; demographical data, such as; sex, age, tribe, level of education, mental status, and occupation.

Questions on healthcare-seeking behaviour consisted of 8 Likert questions with a 5-point scale namely; never 1, rarely 2, sometimes 3, most times 4, and every time 5. Perceived severity and Perceived benefit consisted of 8 items. Perceived benefit was structured by 9 items on a Likert scale of 5 points; strongly disagree 1, disagree 2, neutral 3, agree 4, strongly agree 5). A tool to measure awareness about formal mental treatment consisted of 11 items of multiple-choice questions. The tool was adopted from Pierce.^8^

Data collection was conducted from October 2020 to November 2020. Three (3) research assistants were recruited and trained by the principal researcher.

Research assistants team consisted 2 qualified nurses working in the mental hospital and psychiatric clinic with experience in providing care for patients with mental problems.

### Research Variables and Measurements

The dependent variable of the study was formal mental treatment-seeking behaviour. The Independent Variables were; Caretaker's Social Demographic Characteristics, Patients' Social Demographic Characteristics, Awareness of the caretakers, Perception of the caretakers.

To make variables measurable (operational sing) and meaningful, variables were made operational with precise definitions, scale, and indicators (measurement) to ensure that everyone understands exactly what has been measured for consistency in measurement. For this study, only 3 variables were operational. These were dichotomised or easy two-by-two analysis (Table 1).

**TABLE 1:** Measurement of Variable

Research Variables	Measurement	
	Level of Measurement	Indicator
Health care seeking behaviour	Scale	1-3 Points = Inappropriately 4-5Points = Appropriately
Perceived severity	Scale	1-3Points=Low Severity 4-5points = Severity
Perceived benefit	Scale	1-3Points=Low Benefit 4-5points = Benefit
Perceived barrier	Scale	1-3Points=Low Barrier 4-5points = Barrier
Awareness	Ordinal	1-3Points=Un aware 4-5points = Aware

### Data Analysis Plan

Data processing and analysis was performed using Statistical Package for the Social Sciences (SPSS) software, version 20. Both descriptive and inferential analysis was worn. Data analysis process started with data cleaning to eliminate unusual information, followed by findings on whether the variables were normally distributed or not by use of mutual measures of central tendency and dispersion. To justify the distribution of variables, a normality test of the skew value of the histogram was established. Numerical data was summarised by use of mean and standard deviation while categorical data was summarised by use of frequency and proportions. Categorical variables for baseline characteristics were compared using the Chi-square or Fisher's exact tests. Logistic regression analysis was conducted for the odds ratio to measure the determinants for health-seeking behaviour as well as control identifiable confounders. All variables with a 95% confidence interval which do not contain or cross one or with *p<0.05* were regarded as statistically significant. Findings were presented by using tables and figures.

### Ethical Approval and Consent to participate

Ethical clearance was obtained from the University of Dodoma after being approved by The University of Dodoma Research Ethical Committee with reference number Re: No. MA.84/261/02/208. Permission to conduct this research in the Zanzibar Islands was granted by the office of Second Vice President of Zanzibar. Written informed consent was obtained from each participant.

## RESULTS

### Background Characteristics of Caretakers of Mental Ill Patients

A total of 246 Caretakers were included in the study, 116 (47.2%) had 46 years and above, the lowest age being 19 years and the highest age was 80 years with mean age of 46±11years. Majority of the study participants 152(61.8%) were female. On the side of Educational Status, majority of the participants 118(48%) had Secondary Education. On the side of marital status, majority of the study participants 164(66.7%) were married. Moreover, majority of the participants (85.4%) were self-employed (Table 2).

**TABLE 2:** Socio-Demographic Characteristics of Care Takers (N=246)

Variable	Frequency (n)	Percent (%)
Age Groups		
19-35	65	26.4
36-45	65	26.4
46+	116	47.2
Sex		
Male	94	38.2
Female	152	61.8
Religion of Respondents		
Christian	2	0.8
Muslim	244	99.2
Level of Education		
No Formal Education	55	22.4
Primary Education	62	25.2
Secondary Education	118	48
University/College	11	4.5
Marital Status		
Single	24	9.8
Marriage	164	66.7
Divorced	18	7.3
Widow	40	16.3
Employment Status		
Government Employment	19	7.7
Private Employment	17	6.9
Self-Employment	210	85.4

### Background Characteristics of the Mentally Ill Patients

A total of 246 mentally ill patients were included in the study, out of which 145(58.9%) were in the age group of 21 to 40 years. Majority of the participants 149(66.6%) had primary education and (Table 3).

**TABLE 3:** Socio-Demographic Characteristics of Mentally Ill Patients (N=246)

Variable	Frequency (n)	Percent (%)
Age Group		
1 - 20 years	47	19.1
21 - 40 years	145	58.9
41 - 60 years	50	20.3
61+ years	4	1.6
Sex of the Patient		
Male	117	47.6
Female	129	52.4
Religions of Respondents		
Christian	2	0.8
Muslim	244	99.2
Level of Educational		
No formal education	29	11.8
Primary Education	149	60.6
Secondary Education	68	27.6
Marital Status of the Patient		
Single	83	33.3
Married	46	18.7
Divorced	106	43.1
Widow	11	4.5

### Health Care-seeking Behaviour among Caretakers of Mentally Ill Patients

**FIGURE 2:**
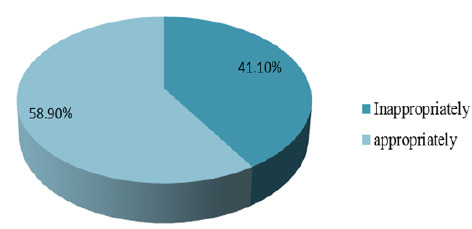
Proportional of Health Care Seeking Behaviour

Majority of the study respondents 145(58.9%) had appropriate health-seeking behaviour towards formal mental treatment while 101(41.1%) of caretakers had inappropriately health-seeking behaviour toward formal mental treatment (Figure 2).

**FIGURE 3:**
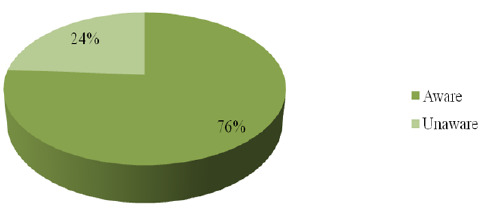
Proportional of Awareness on Formal Mental Health Treatment (N=246)

The findings of this study revealed that majority of study participants 187(76%) had ever heard about formal mental illness treatment while 59(24%) of participants had never heard about formal treatment of mental illness (Figure 3).

### Proportional Distribution of Perceived benefit towards Formal Mental Illness Treatment

The study findings revealed that most of the study respondents 139(56.5%) had perceived benefit towards formal mental illness treatment on mental illness while 107(43.5%) of caretakers had perceived low benefit towards formal mental illness treatment.

### Proportional Distribution of Perceived Barriers toward Formal Mental Illness Treatment

The current study observed that majority of study respondents 198(80.5%) had perceived a low barrier toward formal mental illness treatment while 48(19.5%) of caretakers had perceived a barrier toward formal mental treatment.

### Proportional Perceived severity towards mental illness on health-seeking care behaviour among caretakers of mental illness

Most of the respondents 168(68.3%) had perceived severity towards mental illness on health care seeking behaviour among caretakers of mentally ill patients while 78(31.7%) of caretakers had perceived low severity toward mental illness on healthcare-seeking behaviour among caretakers of a mentally ill patient.

### Relationship between Socio-Demographic characteristics and formal Mental Treatment-Seeking Behaviour among Caretakers of Mentally Ill Patients

Socio-demographic Characteristics of Caretakers were assessed to determine their relationship with healthcare-seeking behaviour. A Chi-Square test was performed to find the relationship. Results show that 4 items exhibits a relationship with healthcare-seeking behaviour. The items include; Perceived low barrier *p <.001*, perceived benefit *p <.008*, perceived severity *p <.001*, and awareness *p<.001* (Table 4).

**TABLE 4:** The Relationship between the Predictors of HBM and Health Care Seeking Behaviour

Variable	Health care Seeking Behaviour		χ2	p-value
	Appropriately (%)	In appropriately n(%)		
Perceived Barrier				
No barrier	132 (66.7)	66 (33.3)		
Barrier	13 (27.1)	35 (72.9.1)	25.014	(< 0.001)
Perceived Benefit				
No benefit	53 (49.50	54 (50.5)	6.929	(< 0.008)
Benefit	92 (66.2)	47 (33.8)		
Age				
19-35	38 (58.5)	27 (41.5)	666	0.0717
36-45	41 (63.1)	24 (36.9)		
46 Above	66 (56.9)	50 (43.1)		
Sex of Participate				
Male	53 (56.4)	41 (43.6)	412	0.521
Female	92 (60. 5)	39.5		
Religion of Respondent				
Christian	0 (0.0)	2 (100)	2.895	0.089
Muslim	145 (59.4)	99 (40.6)		
Level of Education				
No School	33 (60.0)	22 (40.0)	4.436	0.218
Primary Education	30 (48.4)	32 (51.6)		
Secondary Education	74 (62.7)	44 (37.3)		
University or College	8 (72.7)	3 (27.3)		
Marital Status				
Single	13 (54.2)	11 (45.8)	3.709	0.295
Marriage	101 (61.61)	63 (38.4)		
Divorce	7 (38.9)	11 (61.1)		
Widow	24 (60.0)	16 (40.0)		
Awareness				
Aware	126 (86.9)	61 (60.4)	22.932	0.001
Not aware	19 (13.1)	40 (39.6)		
Perceived Severity				
Perceived severity	122 (84.1)	46 (45.5)	40.949	0.001
perceived not severity	23 (15.9)	55 (54.5)		

### Factors Influencing Formal Mental Treatment - Seeking Behaviour

Binary logistic regression analysis was performed to determine factors influencing formal mental treatment-seeking behaviour. The results show that only four variables are associated with healthcare-seeking behaviour. The items are; perceived barriers with health care seeking behaviour OR 5.385 *p<0.00* and perceived severity with health care seeking behaviour OR 6.342 *p<0.00*, awareness of health care seeking behaviour OR 4.349 *p<0.00* and perceived benefit (OR 1.994 *p<0.009*) was statistically significant while all social demographical characteristic of caretakers were not statistically significantly (Table 5).

**TABLE 5:** The Association Between the Predictors of HBM and Health Care Seeking Behaviour among Caretakers of Mental Ill Patients' Simple Binary Logistic Regression

Variable	OR	95% CI		p-value
		Lower	Upper	
Perceived Benefit				
low benefit	1			
Benefit	1.994	1.189	3.344	.009
Perceived Barrier				
Barrier	1			
Low barrier	5.385	2.669	10.863	<.001
Perceived Severity				
Perceived low severity	1			
Perceived severity	6.342	3.504	11.478	<.001
Awareness				
Low aware	1			
Aware	4.349	2.326	8.131	<.001
Age Group				
19-35	1			
36-45	1.066	.576	1.972	.838
46+	1.294	.694	2.414	.418
Sex				
male	1			
Female	0.843	0.500	1.420	.521
Level of Education				
No School	1			
Primary Education	0.563	0.134	2.356	.431
Secondary Education	0.352	0.085	1.451	.148
University or College	0.631	0.159	2.503	.512
Marital Status				
Single	1			
Marriage	0.788	0.283	2.190	.648
Divorce	1.068	0.527	2.166	.854
Widow	0.424	0.136	1.326	.140
Employment Status				
Self Employment	1			
Private Employment	1.039	0.2750	3.919	.955
Government Employment	1.049	0.405	2.715	.922

Multivariate logistic analysis was performed to confounders and result revealed that awareness (AOR 2.907 at 95% CI 2.349-2.326 *p=0.004*) and perceived severity (AOR 4.651 at 95% CI 2,397-9.021 *p<0.000*) were statistically significantly associated with health care seeking behaviour while perceived benefit (AOR 1.25 at 95% C I0.691-2.267 *p=0.46*) and perceived barrier (AOR 1.589 at 95% CI 2.669-10.863 *p=0.295*) were not statistically significantly associated with the health-seeking behaviour (Table 6).

**TABLE 6:** The Association between the Predictors of HBM and Health-care Seeking Behaviour with Multivariate Logistic Regression

Variable	AOR	95% CI		p-value
		Lower	Upper	
Perceived Benefit				
No benefit	1			
Benefit	1.251	0.691	2.267	.46
Perceived Barrier				
Barrier	1			
No barrier	1.589	0.668	3.784	.295
Perceived Severity				
Perceived no severity	1			
perceived severity	4.6	2.397	9.021	.000
Awareness				
Not aware	1			
Aware 2.9	1.406	6.009	.004

## DISCUSSION

The study was conducted to determine the influence of awareness and perception on formal mental treatment seeking behaviour, among caretakers of mentally ill patients in the community of Zanzibar. The study findings revealed that majority of caretakers were aware of formal mental treatment. Despite being aware, majority of them reported having a history of seeking traditional treatments for their patients before seeking for formal mental treatment. A previous study done by Iseselo et al.,^9^ reported that attitude towards psychotropic medication is one of the challenges of formal mental health treatment seeking behaviour. A study conducted in Bangladesh reported that majority of the study respondents were not aware of formal mental health treatment.^24^ This finding is contrary to the findings of this study. The possible reasons for the different findings could be differences in the study respondents' characteristics. This study studied caretakers of mentally ill patients while the study in Bangladesh recruited its respondents from the general population.

Further, the findings from this study showed that patients with chronic mental illness are more likely to be aware of formal treatment compared to patients with acute mental illness. The possible reasons could be due to the prolonged mental illness treatment process. The current finding is contrary to the study conducted by Holden et al^21^, who found that majority of the respondents were not aware of mental illness problems. The difference among respondent it may be due to mass mental health education companies, for example, outreach program, TV and Radio program, school health program and hospital visiting services.

The current study observed that most caretakers had perceived benefits towards formal mental illness treatment but the results reveal that there is no statistical significance between perceived benefit and health care seeking behaviour. This could be due to the fact that caretakers have sufficient knowledge about mentally illness. Similar to the study done by Latunji and Akinyemi^17^ who found that 62% of the respondents perceived mental illness as a chronic disease that can be cured by modern treatment. Also another similar study conducted by Girma and Tesfaye^22^, shows that 98.7% of the respondents believed that mental illness can be cured with modern treatment. This is a bit contrary to what was reported by O'Connor et al.,^23^ where the majority of the respondents perceived mental illness cannot be cured by modern treatment as a long-term treatment. This distinction might be due to differences in study design used and sample size.

In this study, it was found that majority of caretakers had perceived low barrier toward formal mental treatment. This means that caretakers were ready to take responsibility for their patients no matter the consequences and this could probably be due to the reason that they had become aware of the importance of formal mental treatment which made them have the appropriate healthcare seeking behaviour. Similar results from a study conducted in Tanzania^12^ reported that majority of the respondent reported low barriers and had a positive belief about treatment. The current results differ greatly from what was found by a previous study conducted in Zanzibar^9^ which reported that therapeutic could not bring any changes to patient when they failed to see the changes immediately, and thus they were likely to seek alternative treatment, especially from a traditional healer, this finding shows that poor prognosis may lead to barrier on formal treatment. Moreover, the study was contrary to what was reported by Hendersonet al^13^ who found that the most common barrier to formal treatment is a belief that mental illness would go away or would be solved on its own without treatment. Meanwhile, a study by Iyiola^14^ showed strong agreement of the respondent on the perceived barrier to accessing formal treatment.

The current study found that majority of caretakers had perceived severity towards mental illness on healthcare-seeking behaviour. This could be because the caretakers were already having experiences on recurrence and relapse conditions of their patients, that is why they were taking immediate action of use of formal treatment to avoid complications. Similar results were reported from a study conducted by Asfaw et al.,^27^ who found that caretakers had high perceived severity toward mental illness. The finding is similar to the study conducted by Hendersonet al.^13^ The same to the study conducted by Girma and Tesfaye^22^ who found that the majority of the respondents perceived that mental illness is a chronic mental disease that can be cured by modern treatment. The current results differ greatly from what was found by a study conducted in Uganda^11^ where respondents' perceived severity was not a significant predictor of the intention to seek help. This difference might be due to variations in study location, sample size, study design and sample method.

## CONCLUSION

Majority of caretakers of mentally ill patients were aware of formal mental illness treatment. Also, more than half of the caretakers had appropriate healthcare-seeking behaviour. Factors influencing formal mental treatment-seeking behaviour were awareness of formal mental treatment and perceived severity of mental illness. The study recommends a community sensitization campaign to raise awareness and perception towards formal mental health treatment. Community sensitization is crucial for improving formal mental health seeking behaviour.
